# FFGA1 Protein Is Essential for Regulating Vegetative Growth, Cell Wall Integrity, and Protection against Stress in *Flammunina filiformis*

**DOI:** 10.3390/jof8040401

**Published:** 2022-04-14

**Authors:** Muyun Du, Yongbo Xie, Meng Wang, Huan Yang, Banghui Hu, Irum Mukhtar, Yuanyuan Liu, Yongxin Tao, Fang Liu, Baogui Xie

**Affiliations:** 1 Mycological Research Center, College of Life Science, Fujian Agriculture and Forestry University, Fuzhou 350002, China; dumuyun@163.com (M.D.); xbt145978@163.com (Y.X.); wm177208060212021@163.com (M.W.); 17835422047@163.com (H.Y.); hbh1918210324@163.com (B.H.); lyylyy0815@163.com (Y.L.); taoyongxinmuse@163.com (Y.T.); 2 Institute of Soil and Fertilizer, Guizhou Academy of Agricultural Sciences, Guiyang 550006, China; 3 Institute of Oceanography, Minjiang University, Fuzhou 350108, China; erumm21@yahoo.com

**Keywords:** *Flammunina filiformis*, signal transduction, Gαi, RNA interference, overexpression, stress resistance

## Abstract

*Flammulina filiformis* is a popular mushroom which has been regarded as a potential model fungus for mycelium growth, fruiting body development, and stress response studies. Based on a genome-wide search, four genes encoding heterotrimeric G protein α subunits were identified in *F. filiformis*. The data of conserved domain analysis showed that these genes contain only one subgroup I of Gα subunit (Gαi), similar to many other fungi. To explore the function of Gαi, *FfGa1* over-expression (OE) and RNA interference (RNAi) strains were generated using the *Agrobacterium tumefaciens*-mediated transformation (ATMT) approach. RNAi transformant strains showed remarkably reduced growth on PDA medium and added sensitivity to cell wall-enforcing agents with maximum growth inhibition, but showed better growth in response to hypertonic stress-causing agents, while OE strains exhibited more resistance to thermal stress and mycoparasite *Trichoderma* as compared to the wild-type and RNAi strains. Taken together, our results indicated that *FfGa1* positively regulates hyphal extension, and is crucial for the maintenance of cell wall integrity and protection against biotic and abiotic (hypertonic and thermal) stress.

## 1. Introduction

*Flammulina filiformis* is one of the most widely cultivated mushrooms with nutritional and medical values [1,2]. To date, extensive research has shown that mushroom growth is mainly affected by temperature, moisture, light, carbon dioxide concentration, pathogenic microorganisms, and other environment factors [3,4]. However, *F. filiformis* is more sensitive to high temperatures, resulting in strong negative effects on hyphal extension, primordium formation, and fruiting body yield when compared with *Pleurotus ostreatus*, *Lentinula edodes*, and *Volvariella volvacea* [3,5]. Owing to the important progress in multiple omics research and RNA interference and overexpression systems construction [5,6,7], together with the characteristics that obvious phenotypic traits between vegetive growth and fruiting body development, and sensitivity to environmental stimuli, *F. filiformis* has been regarded as a potentially excellent model for macro-fungal genetics, development, and environmental stress response studies [2,6].

Heterotrimeric G protein is critically important in transduction extracellular stimuli into intracellular signaling pathways in almost all eukaryotes [8,9]. It is composed of Gα, Gß, and Gγ subunits conserved in fungi; its classification is mainly based on types of Gα subunits [10]. Gα subunits were classified into three distinct groups in most fungi, which obtained the group I subfamily (related to the mammalian Gαi superfamily), group II subfamily, and group III subfamily (related to the mammalian Gαs superfamily) [11]. Different Gα subunits show diverse or overlapping functions in the same species [11,12,13]. For example, there are three Gα encoding genes, *gna-1*, *gna-2*, and *gna-3*, in *Neurospora crassa* [11]; deletion of *gna-1* or *gna-3* causes a defect in sexual life cycles [14,15], but the absence of *gna-2* has no obvious effect on growth and development [16]. What’s more, three Gα subunits induce hyphal growth, but have various effects on gliotoxin production in *Aspergillus fumigatus* [17].

Gαi subunit is important for mycelium growth, development, and the stress response in fungi. Gαi has a positive effect on conidial germination, thermal tolerance, and cell wall integrity in *Metarhizium robertsii* [18], but has the opposite effect on thermal stress in *N. crassa* [19]. In addition, Gαi is involved in antagonism against *Sclerotium rolfsii* in *Trichoderma virens* [20]. However, the function of Gαi subunits in *F. filiformis* remains unknown. In the present study, four genes (*FfGa1-4*) encoding Gα subunits were identified in *F. filiformis*, which were classified into three distinct groups. Among them, we found that only the FFGA1 mRNA level was significantly increased in mycelium stage when compared with all the fruiting body development stages. Therefore, *FfGa1* was selected to explore its roles in vegetative stage of *F. filiformis*. The data of this study are beneficial for investigating the influence and mechanism of G proteins on vegetative growth and stress response in macro-fungi.

## 2. Materials and Methods

### 2.1. Fungal Strains and Culture Conditions

The wild-type *F. filiformis* dikaryotic strains FL19 and mononuclear strain L11 (mononucleate protoplast from FL19) and *Trichoderma* sp.0018 (mycopathogen) were obtained from the Fujian Edible Fungi Germplasm Resource Collection Center of China. The strain L11 and transformants were maintained on potato dextrose agar (PDA) or complete yeast medium (CYM); plasmid propagation of *Escherichia coli* (DH5α) and *Agrobacterium tumefaciens* (GV3101) were incubated in lysogeny broth (LB) containing ampicillin (100 μg/mL) or kanamycin (50 μg/mL); co-cultured wild-type L11 and *A. tumefaciens* were inoculated on induction medium (IM) which included 10 mM glucose, 10 mM K_2_HPO_4_, 10 mM KH_2_PO_4_, 0.7 mM CaCl_2_, 2 mM MgSO_4_·7H_2_O, 9 μM FeSO_4_·7H_2_O, 2.5 mM NaCl, 4 mM (NH4)_2_SO_4_, 0.5%(*w*/*v*) glycerol, 200 μM acetosyringone(AS), and 40 mM 2-(N-Morpholino)ethanesulfonic acid(MES)(pH5.3), as previously described [21,22]. The fruiting bodies of dikaryotic FL19 strains were cultivated on the medium as Wang et al. described [23].

### 2.2. Phylogenetic Analysis of Gα Sequences

To identify and compare the Gα subunit in *F. filiformis*, the Gα protein sequences of *S. commune* were used as queries for the BLASTP search against the predicted protein sequence database of the L11 genome (not published). A phylogenetic tree was constructed with the MEGA6 program using the neighbor joining method with a 1000 bootstrap value [24]; the motif of Gα proteins was predicted using the MEME website (http://meme-suite.org/tools/meme, accessed on 16 March 2021).

In order to analyze sequence conservative structural characteristics, the amino acid sequences were aligned in Clustal W, domain functions were searched in the National Center of Biotechnology Information (NCBI), the phyre2 (http://www.sbg.-bio.ic.ac.uk/phyre2/html/page.cgi?id=index, accessed on 16 March 2021) was used to predict the protein structure, and the final result was visualized with GeneDoc (Version 2.7) after manually editing.

### 2.3. Generation of Overexpression and Knockdown Strains

A specialized pBHg-BCA1 transformation vector [1] with the hygromycin B phosphotransferase (*Hpt*) gene as a selectable marker and glyceraldehyde-3-phosphate dehydrogenase (*Gpd*) gene promoter (found in the Mycological Research Center of Fujian Agriculture and Forestry University, Fuzhou, China) was used for *FfGa1* gene knockdown (RNAi) and overexpression (OE) vector construction by replacing the *SpeI*-*ApaI* fragment BAC1 with the target fragment.

For the construction of overexpression vector *FfGa1*-OE, the full-length open reading frame of the *FfGa1* gene was amplified from L11 gDNA using primer pairs (*VFfGa1*-OE-F/R) with added *SpeI* and *ApaI* sites. The PCR product was digested with *SpeI* and *ApaI*, and then ligated into the pBHg-BCA1 plasmid, while the *FfGa1*-RNAi vector fragment consisted of two parts to form the hairpin; the left fragment contained the 4th exon and 3rd intron, and the right fragment contained the 4th exon in an opposite orientation; two parts were amplified by PCR individually with *SpeI*/*ApaI* restriction sites using the primers (*VFfGa1*-RNAiL-F/R and *VFfGa1*-RNAiW-F/R), and then were introduced into pBHg-BCA1 vector driven by the endogenous *Gpd* promotor to generate the *FfGa1*-RNAi vector.

### 2.4. Fungal Transformation and Screening of Positive Transgenic Strains

*F. filiformis* L11 strain was transformed by plasmids *FfGa1*-RNAi and *FfGa1*-OE using the *Agrobacterium tumefaciens*-mediated transformation approach (ATMT) [25,26] with a slight modification in the method, as mycelial plugs from the edge of the L11 colony were transferred into centrifuge tubes together with *A. tumefaciens* in liquid IM for 6 h; after inoculation, co-cultures were maintained on solid IM medium covered with sterile cellophane at 25 °C for 4 days; in order to remove the *A. tumefaciens* as cleanly as possible, co-cultures were rinsed in a 50 mL sterile tube, which contained 40 mL of sterile water added to 200 ug/mL cefotaxime; finally, mycelial plugs were dried with sterile filter paper and then cultured on CYM medium supplemented with 30 µg/mL hygromycin and 100 µg/mL cefotaxime at 25 °C.

All putative transformants were first selected on PDA plates containing hygromycin B (50 μg/mL) five times to stabilize the genotype for further use. For molecular confirmation, total genomic DNA was extracted using a modified cetyltrimethylammonium bromide (CTAB) method from the 7 day-old mycelium of each putative transformant, grown separately on cellophane overlaying PDA at 25 °C without hygromycin. Isolated DNA was used as a template for PCR amplification. Positive transformants of *FfGa1*-OE and *FfGa1*-RNAi were verified using the forward primer GBT-F (from the *Gpd* promoter) and the reverse primers *VFfGa1*-OE-R and *VFfGa1*-Ri-R (for the *FfGa1* gene silencing fragment: a part of the *Gpd* promoter, 4th exon, and 3rd intron) to amplify the fragment in separate PCR amplifications. Whole-genome sequencing of transformants was conducted to analyze vector insert sequence and estimate the copy number of insert fragment. Resequencing reads mapped on OE/RNAi vector and genome were extracted to analyze vector insert sequences via Burrows–Wheeler Aligner (BWA version 0.7.17) as previously described [27]; the sequence depth of vector and genome were calculated by SAMtools Version 1.9, and then the copy number of insert was counted by the ratio of average sequence depth of genome and insert fragments [28].Total RNA was also isolated from the mycelium of putative transformant strains to measure the expression levels of *FfGa1* by quantitative real-time polymerase chain reaction (qPCR).

### 2.5. Phenotypic Characterization of OE and RNAi Transformants

To investigate the growth rate of the OE and RNAi strains, hyphal plugs (4 mm) were obtained from the growing periphery of the wild-type (WT) and transformants colonies, inoculated separately onto PDA medium. Growth-initiation lines were drawn based on a cross line with the hyphal plug as the center on the 3rd day, and growth-termination lines were drawn on the 6th day. Apical extension rate = distance (growth-initiation line to growth-termination line)/3 d.

To measure mycelial dry weight biomass, the WT and the transformants were cultured into liquid PDB medium, which was PDA medium without agar, incubated in a dark chamber at 25 °C. After 10 days, mycelium was collected by sterilized sieves, then washed 3 times using sterilized distilled water, pressed between the filter papers to remove water, and dried at 85 °C for at least 12 h.

For the sensitivity test, the WT and transformants were inoculated on PDA medium supplemented with hypertonic stress agents 0.3 M NaCl and 0.5 M KCl, and the cell wall-perturbing agents were supplemented with 0.01% sodium dodecyl sulfate (SDS), 200 ug/mL Congo red, or 200 ug/mL Calcofluor white (CFW), in the dark at 25 °C for 6 days. For thermal stress, strains were maintained on PDA medium for 3 days in the dark at 25 °C and then transferred to 30 °C for 3 to 7 days in the incubators for the thermal stress assay. Inhibition ratio = 100% − (the growth rate of untreated strain − the growth rate of treated train) × 100%.

### 2.6. Resistance of F. filiformis Transformants against Trichoderma

To assess the resistance of *F. filiformis* transformants against pathogens, the *Trichoderma* sp.0018 strain was employed for a pathogenicity assay using the dual culture technique. A mycelial plug (4 mm) of *Trichoderma* sp.0018 was placed on medium 3 cm apart from the margin of the plate. However, a WT/OE/RNAi mycelial plug was inoculated on the same medium plate 4 days prior to *Trichoderma* sp.0018 inoculation, due to the slow growth of the WT/OE/RNAi strain when compared to *Trichoderma* sp.0018. The effect of the co-cultivation of fungal strains was determined for 3 days. In another assay, *Trichoderma* culture filtrate was used for the resistance of *F. filiformis* transformants against *Trichoderma* [29]. For the preparation of culture filtrate, mycelium plugs of *Trichoderma* sp.0018 were inoculated into 100 mL liquid PDB medium in a flask and placed into an incubator shaker (150 rpm) at 25 °C for 3 days. After incubation, PDA medium was prepared with culture filtrate, and different concentrations of culture filtrate were used to assess the effect on the growth of *F. filiformi* (WT/OE/RNAi) strains in the dark at 25 °C for 6 days.

### 2.7. RNA Isolation, Complementary DNA (cDNA) Synthesis, and Expression Analysis of FFGA, Hydrophobin, and Chitin Synthetase

Total RNA from the transformants was isolated using an E.Z.N.A.™ Plant RNA Kit (Omega, Stamford, CT, USA) according to the manufacturer’s protocol and quantified using a NanoND-1000 spectrophotometer (NanoDrop Technologies, Wilmington, DE, USA). For each sample, 1000 ng of RNA sample was reverse transcribed into a total volume of 20 μL of cDNA using TransScript^®®^ One-Step gDNA Removal and cDNA Synthesis SuperMix (TransGen Biotech, Beijing, China), random primers, and an oligo dT primer base.

To measure the expression levels of FFGA, as well as hydrophobin and chitin synthetase, quantitative PCR was performed on a CFX96 Real-Time PCR Detection System (Bio-Rad, Hercules, CA, USA) using the Transcript^®®^ Tip qPCR SuperMix (TransGen Biotech, Beijing, China) according to the manufacturer’s instructions. Three amplifications with three technical replicates were conducted for each sample. All amplifications included a denaturation step for 10 s at 95 °C, followed by 40 cycles of 5 s at 95 °C and 30 s at 58 °C. *RAS* and *GAPDH* were used as reference genes for the normalization of the qPCR data [30]. All primers in the study (Supplementary Table S1) satisfied the requirement that Sean Taylor mentioned in RT-qPCR MIQE [31]. The expression of the genes was analyzed using the 2^−ΔΔ*C*t^ method [32].

### 2.8. Statistical Analysis

Three biological replications with technical replicates were employed for each experiment. Statistical analysis was performed using SPSS version 25.0. The statistical significance was evaluated by one-way ANOVA in combination with LSD’s multiple-comparison test, using the different lower case to express significant differences among samples (*p* < 0.05) [33]. The results were visualized into heat maps by TBtools version 1.055 and histograms of the means ± standard deviations by GraphPad Prism version 7.02.

## 3. Results

### 3.1. Identification and Characterization of Gα Subunit

We identified four putative genes, *FfGa1* (OK156002), *FfGa2* (OK156003), *FfGa3* (OK156004), and *FfGa4* (OK156005), encoding Gα subunits in the *F. filiformis* genome, which encode the polypeptide ranging from 339 to 355 amino acids in length. The products of the four genes shared 33.6 to 55.56% identity, along with 40.28 to 86.22% identity to ScGP-B from *S. commune*.

**Figure 1:**
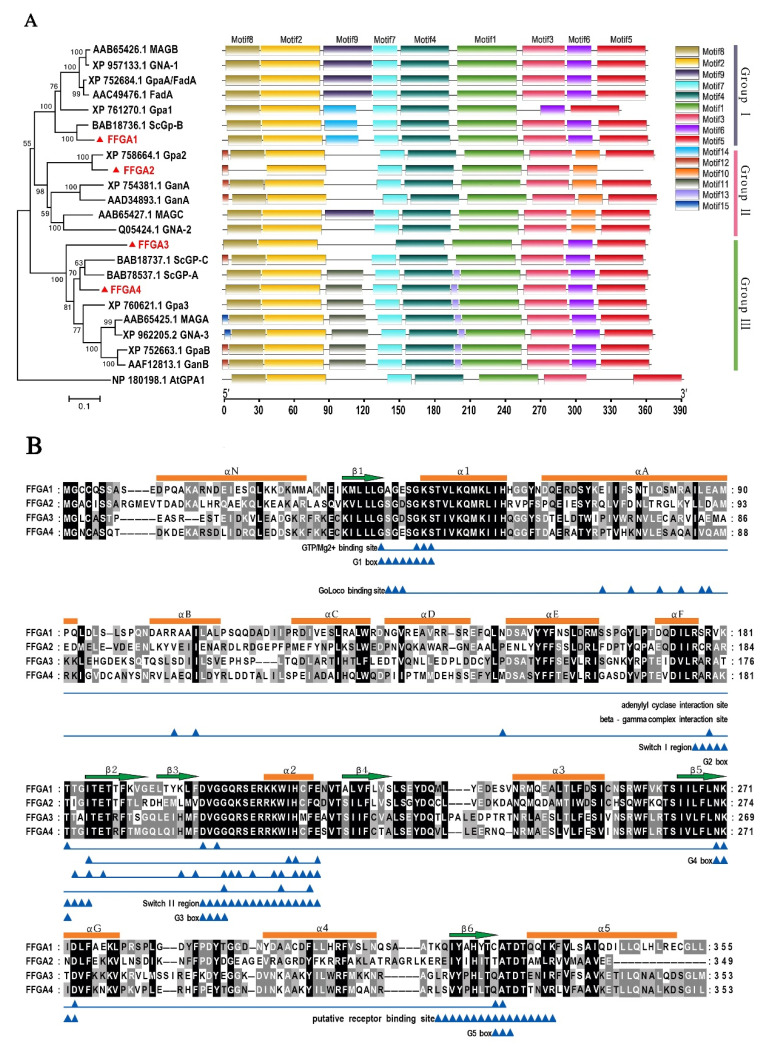
Analysis of Gα amino acid sequences. (**A**) Phylogenetic analysis of FFGA and the Gα protein from other fungi. The phylogenetic tree was constructed using MEGA software version 6 with a bootstrap method of 1000 replications. Conserved motifs marked in different colors and numbers were generated by MEME tool. Motif 1 contains the G3 box and Switch II region; Motif 2 contains the G1 box; Motif 3 contains the G4 box; Motif 4 contains the G2 box and the Switch I region; Motif 5 contains the G5 box. (**B**) Multiple sequence alignment and secondary structure features of FFGA1-4. There is a pertussis toxin CXXX (located in C terminus) and cholera toxin RSRVK (located in switch I) in FFGA1. Black background with white letters represents the high-identity region in the amino acid sequence, while secondary structure (α helices and β-sheet) ruler numbering was based on FFGA1.

In order to determine the evolutionary relationship of the identified FFGA1-4 in *F. filiformis* with Gα ortholog sequences reported in other fungal species (i.e., *Aspergillus nidulans* (AAD34893.1, BAB78537.1 AAF12813.1), *Aspergillus fumigatus* (XP_752684.1, XP_754381.1 and XP_752663.1), *N. crassa* (XP_957133.1, Q05424.1 and XP_962205.2), *Magnaporthe grisea* (AAB65426.1, AAC49476.1, AAF12813.1), *Ustilago maydis* (XP_761270.1, XP_758664.1, XP_760621.1), and *S. commune* (BAB18737.1, BAB78537.1, BAB18736.1)), and a detailed phylogenetic analysis and conserved motif comparison were performed. The phylogenetic tree showed three prominent clades, which correspond to the three major groups described in a previous study [34]; all proteins had the Motif 1–5 presenting the conserved G1-5 box of G protein, and displayed strong variations in the motif of 90aa–130aa (Figure 1A). FFGA1 of *F. filiformis* showed a high degree of identity and motif similarity with BAB18726.1 of *S. commune*, which belongs to the Gα group I subfamily (related to the mammalian Gαi superfamily), and FFGA2 exhibited a closer association with Gα subunits in group II, while both FFGA3 and FFGA4 were clustered into group III.

Pfam-based domain prediction (http://pfam.janelia.org, 18 March 2021) revealed the presence of highly conserved guanine nucleotide-binding sites in four FFGA proteins, which is a key domain for G-protein subunit identification. Furthermore, there was a conserved protein fold among Gα proteins consisting of the Ras-like domain (RasD) and Helix domain (HD) [10], as shown in Figure 1B. RasD is a six β-sheet (β1–β6) surrounded by five α-helices (α1–α5), which is a highly conserved sequence in G1-G5 box, as reported by Yao [35], and is involved in the distinctive characteristics of G protein, including the GTP binding motif “GXGESGKS” (located in G1) of Motif 2, GTPase domain “DXXGQ” (located in switch II) of Motif 4, and myristoylation (MGXXXS) [10,32]. Therefore, the protein sequence analysis indicated that four Gα subunits are classical Gα proteins, whereas FFGA1 showed the two conserved functional motifs of the Gαi subunit, canonical ADP-ribosylation sequences for cholera toxin “RSRVK” (located in switch I), and ADP-ribosylation site for pertussis toxin “CXXX” (located at its C terminus), thus further confirming that FFGA1 belongs to the Gαi subfamily (Figure 1B).

### 3.2. Expression of Gα Subunit at Different Stages of F. filiformis

**Figure 2:**
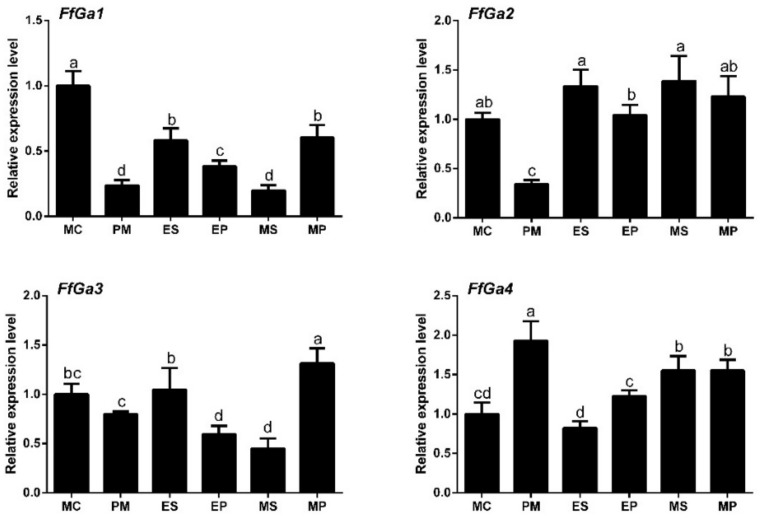
*FfGa* gene expression patterns during different development tissues. The expression was monitored at MC: mycelium; PM: primordium; ES: elongation stage of stipes; EP: elongation stage of pileus; MS: mature stage of stipes; MP: mature stage of pileus. The *GAPDH* and *RAS* genes were used as the reference genes for evaluation of the expression. The expression at the mycelium stage was assigned a value of 1 for normalization. The letters “a”, “b”, “c”, and “d” indicate statistically significant difference at 0.05 level between samples. Bars with no common letters are significantly different (*p* < 0.05).

To explore the possible relationship between the expression level of *FfGa1-4* and morphological growth, *FfGa1-4* transcript accumulation on different tissues, including mycelium (MY), elongation-stage stipes (ES), mature-stage stipes (MS), elongation-stage pileus (EP), and mature-stage pileus (MP), was assessed. The expression at the mycelial stage was used as a control, and it was assigned an expression value of one for normalization. Our results showed that the mRNA abundance of Gα genes was low in PM, except for *FfGa4* (Figure 2). When compared with MC, the *FfGa1* mRNA level was significantly reduced at all the stages tested with fold decreases of −0.7, −0.4, −0.6, −0.8, and −0.4 at PM, ES. EP, MS, and MP stages, respectively; the *FfGa2* transcript level was slightly increased in stipes and pileus, a similar result to that shown in *FfGa4*, while the expression level of *FfGa3* showed a weak reduction in PM, EP, and MS, and exhibited a distinct difference between elongation pileus and mature pileus. The result showed that the expression of *FfGa1* in all parts of fruiting bodies was lower than that of mycelium, whereas the expression of *FfGa2-4* presented no regularity between mycelium and several fruiting body tissues. Therefore, we could generate the OE and RNAi transformants and analyzed their respective phenotypes to explore the exact roles of the *FfGa1* on the vegetative stage.

### 3.3. Generation of Overexpression and Knockdown Transformants

**Figure 3:**
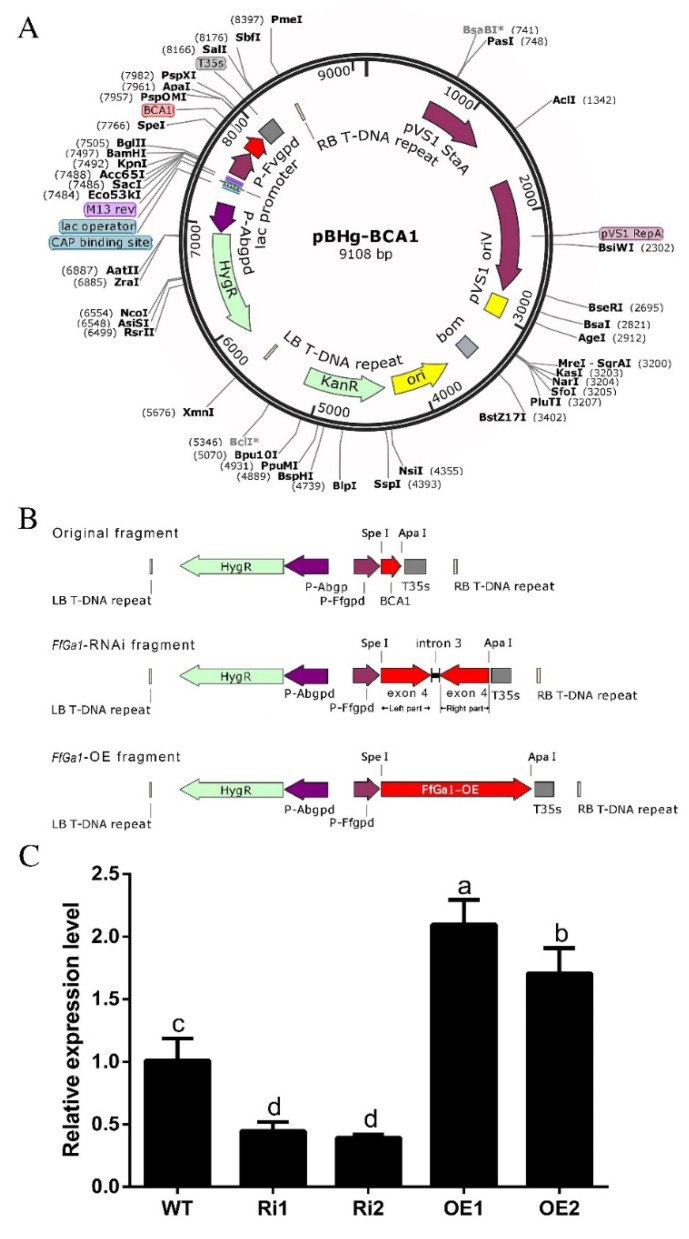
Construction strategy of OE and RNAi vectors and transformants screening. (**A**) Vector map representing original plasmid pBHg-BCA1 used for the generation of transformants. (**B**) Cloning strategy showing the RNAi and OE cassette employed in generation of transformants. (**C**) *FfGa1* expression in transformants. The expression of *FfGa1* in WT was set as a value of 1. The meaning of “a”, “b”, “c”, and “d” see Figure 2 legend.

*FfGa1*-OE and RNAi vectors were constructed based on the binary vector pBHg-BCA1 mediated by *A. tumefaciens* (Figure 3A,B). Putative *FfGa1*-OE and RNAi transformants were screened by PCR with primer pairs (Supplementary Table S1) for *FfGa1*-OE and RNAi transformants. The results from PCR confirmation assays showed that the target gene fragments were stably inserted in the WT strain (Supplementary Figure S1). To access the efficiency of *FfGa1* gene silencing and overexpression, we performed qPCR analysis to check the expression of *FfGa1* in the transformants. Our qPCR results showed that the transcript levels of *FfGa1* in the OE1 and OE2 transformants were up-regulated with an approximate fold increase of 2- and 1.6-fold higher, respectively, than in the WT strain, whereas *FfGa1* transcription in RNAi transformants was decreased by more than 50% (Figure 3C). In addition, the almost complete LB-RB sequence with one copy was identified in four transformants by the whole-genome sequencing approach (Supplementary Table S2). Therefore, these transformants were chosen for further study.

### 3.4. FfGa1 Positively Regulates Vegetative Growth in F. filiformis

**Figure 4:**
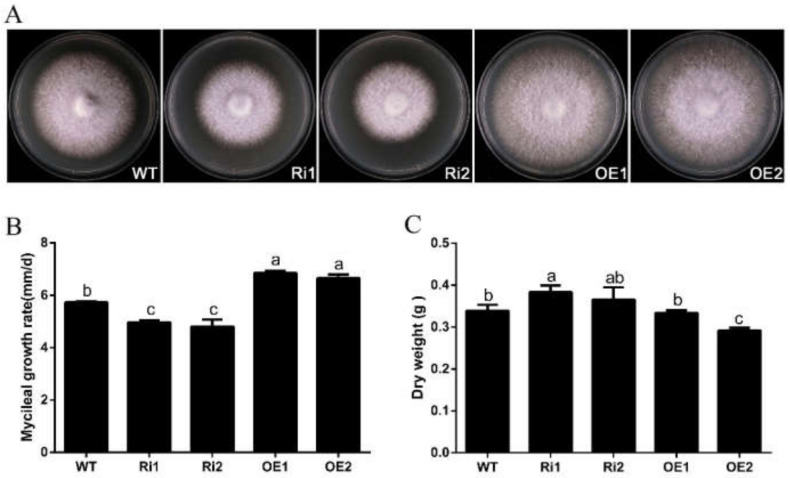
*FfGa1* is required for vegetative growth. (**A**) Colony morphology of WT and transformants cultured on PDA medium and photographed after 6 days inoculation. (**B**) The growth rates of WT, RNAi, and OE strains cultured on PDA. (**C**) Biomass of the WT and transformants cultured in PDB medium for 10 days. See Figure 2 legend for explanation of “a”, “b” and “c”.

The Gα subunit was reported to positively regulate vegetative growth in the fungus *Penicillium camemberti* [12]. To understand the physiological function of *FfGa1* in the growth of *F. filiformis*. The WT, RNAi, and OE strains cultured on PDA medium for 6 days and hyphal growth rate and morphology were monitored. When compared to the WT strain, the OE strains exhibited a significantly increased growth, while the RNAi transformants showed a reduction in growth rate with compact, fluffy, more dense aerial hyphae (Figure 4A,B). We further measured the mycelial dry weight of WT, RNAi, and the OE strains maintained on PDB medium. This showed that mycelial dry weight accumulation was slightly higher for the RNAi strains when compared to the WT strain (Figure 4C). Based on this result, we conclude that *FfGa1* positively regulates hyphal extension, but the knockdown of *FfGa1* could slightly increase the mycelial weight of *F. filiformis*.

### 3.5. FfGa1 Facilitates Thermoresistance

**Figure 5:**
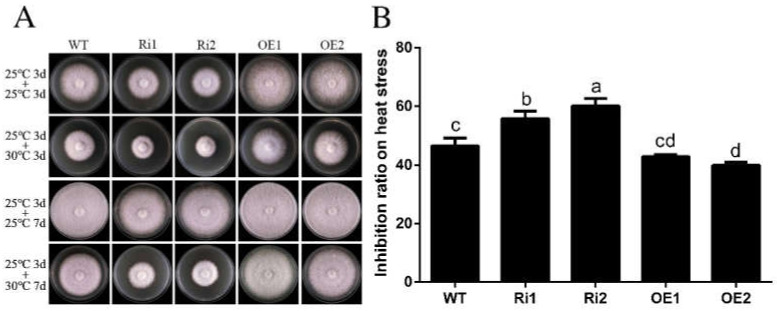
*FfGa1* is required for regulation of thermal stress response. (**A**) The difference of colony morphology and diameter of strains on PDA medium treated at temperatures of 25 and 30 °C. Photographs were taken after 3 and 7 days. (**B**) Statistical representation of the inhibition rates at 30 °C from WT and transformants. See Figure 2 legend for explanation of “a”, “b” “c” and “d”.

A previous study showed that the PGa1 was involved in the regulation of heat stress response in the fungus *Penicillium chrysogenum* [36]. To explore the potential role of the FFGA1 subunit in the thermal stress response in *F. filiformis*, PDA plates inoculated with WT, RNAi, and OE strains were firstly incubated at a normal temperature of 25 °C; three days later, some of the WT, RNAi, and OE strain plates were transferred into a different incubator with a high temperature of 30 °C for 3 days, and the growth rate was measured. In order to obtain a more visible phenotypical difference, the treatment time was prolonged for an additional 4 days to take another photo. Results showed that the RNAi strains were more sensitive to chronical heat stress in comparison to WT, while the OE strains showed high tolerance to thermal stress with maximum colonies in the lower inhibition ratio (Figure 5). Therefore, these results indicate that *FfGa1* has a positive impact on thermal resistance in *F. filiformis*.

### 3.6. FfGa1 Is Required for Maintenance of Cell Wall Integrity and Hypertonic Stress Response in F. filiformis

The resistance to SDS, Congo red (CR), and CFW is considered as a barometer, reflecting cell wall integrity in fungi [37]. To establish the role of *FfGa1* in cell wall integrity in *F. filiformis*, we compared the growth rates of the WT, RNAi, and the OE strains on PDA medium supplemented with cell wall-perturbing agents. The data showed that the growth rates of all strains were decreased on treated PDA, and *F. filiformis* was more sensitive to SDS than CR and CFW. On the other hand, OE transformants were more tolerant to SDS at a lower inhibition ratio with respect to WT; on the contrary, the growth rate of RNAi transformants was reduced severely upon SDS treatment when compared to wild-type transformants, and a similar pattern was observed when strains were treated with Congo red or CFW.

**Figure 6:**
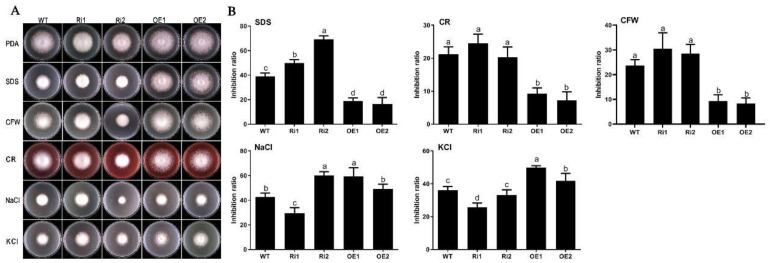
Growth response of WT and transformants under cell wall stress and hypertonic stress. (**A**) Morphology of WT and transformants cultured on PDA medium supplemented with cell wall stressors, i.e., 0.01% SDS or 200 µg/mL Congo-Red (CR), while 0.3 M NaCl or 0.5 M KCl were used as osmotic stress causing agents for six days. (**B**) Mycelial growth inhibition ratio of WT and transformants under cell wall stress or hypertonic stress. See Figure 2 legend for explanation of “a”, “b” “c” and “d”.

We further tested whether *FfGa1* could be involved in response to hyperosmolarity in *F. filiformis* by culturing the WT, RNAi, and the OE strains on PDA medium supplemented with hypertonic agents 0.3 M NaCl and 0.5 M KCl. The results showed that OE transformants were more significantly inhibited at 0.5 M KCl than WT, and the opposite phenotypes of all RNAi transformants (Ri1 and Ri2) had a lower inhibition ratio than OE transformants (Figure 6). Under NaCl stress, OE transformants exhibited a higher inhibition effect than WT; Ri1 transformant had a lower inhibition ratio than OE transformants, while a higher inhibition phenotype was observed in Ri2 transformants, yet the mechanism of different response between Ri1 and Ri2 was unknown. In conclusion, these results suggest that *FfGa1* is favorable to the maintenance of cell wall integrity and involved in the regulation of hypertonic stress responses in *F. filiformis*.

### 3.7. FfGa1 Involved in Resistance to Trichoderma sp.0018

**Figure 7:**
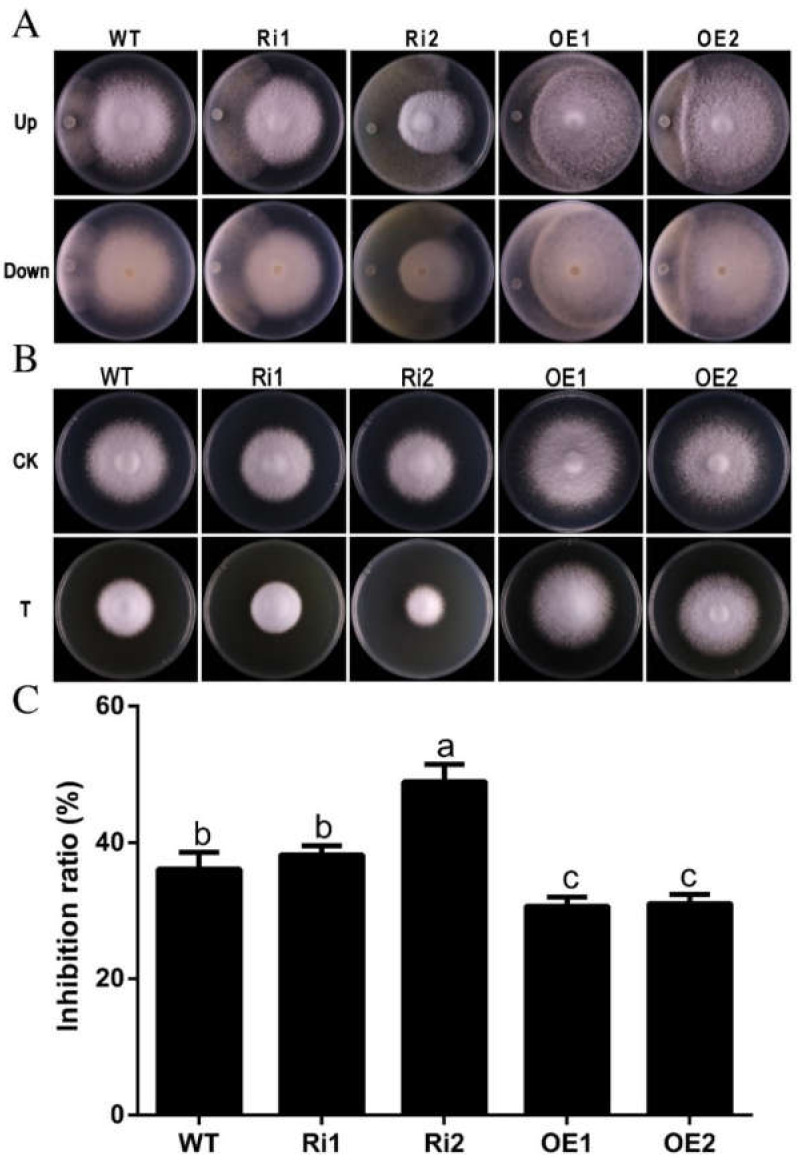
*FfGa1* regulates the resistance to *Trichoderma* sp.0018 in WT, RNAi, and OE. (**A**) WT and transformants’ antagonism against *Trichoderma* sp.0018. The top represents a photo taken from the front-side plate; the bottom represents a photo taken from the reverse-side plate. (**B**) Colony phenotype of WT and mutant strains cultured on PDA medium prepared with or without *Trichoderma* sp.0018 fermentation broth. T represents PDA medium prepared with *Trichoderma* sp.0018 fermentation broth, CK represents PDA medium. Strains were cultured for 6 days then plates were photographed. (**C**) Statistical analysis of mycelial growth inhibition ratio of WT and transformants cultured on PDA medium prepared with or without *Trichoderma* sp.0018 fermentation broth. See Figure 2 legend for explanation of “a”, “b” and “c”.

*Trichoderma* is a well-known mycopathogen that can reduce mushroom production seriously [38]. To evaluate the role of *FfGa1* in *F. filiformis* against *Trichoderma* sp.0018, the dual culture method was applied. Plate antagonistic assay results showed that OE1 and OE2 transformants exhibited obvious resistance against *Trichoderma* by forming a prominent zone of interaction, while no distinct zone of interaction was observed as a result of the co-cultivation of WT or RNAi strains with *Trichoderma* sp.0018 (Figure 7A).

We have also assessed the resistance of *F. filiformis* transformants against *Trichoderma* sp.0018 through culture filtrate assy. PDA medium supplemented with 50% *v*/*v* *Trichoderma* sp.0018 culture broth was used as treatment based on the preliminary experiment (Supplementary Figure S2). Results obtained showed the growth of RNAi strain was highly inhibited on *Trichoderma* sp.0018 culture broth medium, while the OE transformants were more resistant and grew faster when compared with WT and RNAi transformants (Figure 7B,C). These results suggest *FfGa1* plays an important role in the defense mechanism of *F. filiformis* against *Trichoderma* sp.0018.

### 3.8. FfGa1 Regulates the Expression of the Hydrophobin and Chitin Synthase Genes

**Figure 8:**
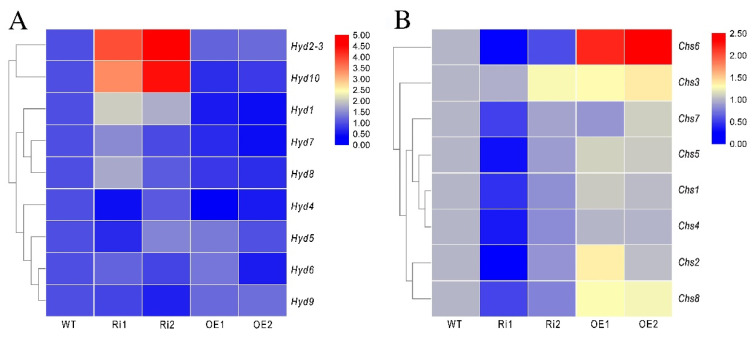
Expression of hydrophobin and chitin synthase genes in transformants. (**A**) Transcript levels of hydrophobin genes in transformants. (**B**) Transcript levels of chitin synthase genes in transformants. The expression of genes in WT was set as a value of 1, the results were visualized by TBtools.

Since the Gα subunit is involved in the regulation of hydrophobin expression in previous studies [39,40], we performed quantitative PCR (qPCR) to test the effects of *FfGa1* on the transcription of the hydrophobin genes *Hyd1*, *Hyd2-3*, *Hyd4*, *Hyd5*, *Hyd6*, *Hyd7*, *Hyd8*, *Hyd9*, and *Hyd10*. Our results showed that the majority of these genes, especially *Hyd2-3*, *Hyd10*, and *Hyd1*, were up-regulated in the RNAi transformants, while in OE transformants, *Hyd10* and *Hyd1* were down-regulated significantly (Figure 8A).

According to the literature, chitin synthase genes are related to cell wall integrity stress, and *Chs5* or *Chs7* deletion of *Fusarium verticillioides* increases susceptibility to SDS stress and exhibits reduction in growth [41]. Therefore, we also performed quantitative PCR (qPCR) to examine the expression of eight chitin synthetase genes. Almost all of these genes were down-regulated more or less in the RNAi strains, and the mRNA level of *CHS6* was increased obviously in OE transformants. This indicates that *FfGa1* favors the mRNA accumulation of most chitin synthetase genes.

## 4. Discussion

Gα protein, as a crucial signal transduction component, has been shown to be indispensable in many biological functions [42]. A variety of Gα subunits usually imply distinct or overlapping function in some organisms, such as *Aspergillus flavus* [43], *Saccharomyces cerevisiae* [44,45], and *Fusarium oxysporum* [46]. In contrast to the majority of filamentous fungi studied to date, which contain three genes that encode Gα proteins [47], four genes encoding Gα proteins were identified based on a genome-wide search of *F. filiformis*; corresponding protein sequences were clustered into three distinct well-characterized groups, and only FFGA1 had a higher transcript level in mycelium than fruiting body tissues.

In order to explore the function of *FfGa1*, RNAi and overexpression techniques were used to generate transformants for phenotypic assays. The results showed that the *FfGa1* positively regulated hyphal extension, since compared to the WT, OE transformants exhibited an increase in growth rate with a larger colony diameter, and reverse phenotypic characteristics were observed in RNAi transformants. This result is consistent with previous studies [11]. In addition, we found that the knockdown *FfGa1* led to a reduction in the growth rate of PDA but showed a minor increase in mycelium accumulation in PDB. In agreement with the role of Gαi of *F. filiformis* in hyphal growth, the deletion of *cpg-1* resulted in a greatly reduced radial growth rate with scarce aerial hyphae, and a higher dry weight when compared with wild-type *Cryphonectria parasitica* [39]. Similar studies carried out in *P. chrysogenum* showed different results, in which Pga1 presented a strong negative effect on apical extension, but had no influence on biomass [48]. However, in this regard, there is a rough correlation between aerial hyphae and dry weight in *N. crassa*, as constitutively active Gαi strains exhibited a higher growth rate, more aerial hyphae, and a greater mass [19]. In light of these evidences, one plausible explanation is that the Gα may play different roles in hyphal growth due to culture conditions and specific species.

A close negative relationship between the Gα subunit expression level and the adaptability to environmental stimuli was verified in *P. chrysogenum* [36]. However, diverse results are found in our experiments showing that the overexpression *FfGa1* increase the susceptibility to hypertonic stress, but is beneficial in enhancing hyphal tolerance to chronic high temperature and maintaining cell wall integrity. Similarly, TrGpa1 positively regulates the adaptability to thermal stress in Basidiomycota *Tremella fuciformis* [49]. In contrast to the negative influence of Gαi on hyperosmolarity in *P. chrysogenum*, the disruption of Gαi strains was less tolerant to hypertonic stress in *N.crassa* [50] and *Cochliobolus heterostrophus* [51]. Therefore, these findings, together with the previous research, highlight the important role of the Gαi subunit in regulating stress response in fungi.

*Trichoderma*, well known as a biocontrol agent against plant pathogens [52], is an harmful pathogen for mushroom cultivation as it suppresses mycelial growth and fruiting body production [38,53]. When confronted with *Trichoderma* sp., there is no distinguishable phenotypical trait in the contact zones for *Agaricus bisporus*, which could result in almost 100% crop loss [54]. Similar results regarding the response to *Trichoderma* sp.0018 attack were obtained in WT and RNAi transformants of *F. filiformis*. Interestingly, there are physiological responses to form mycelium assemblage barriers against the invasion of *Trichoderma* in OE transformants, with a lower inhibition ratio on the PDA medium amended with *Trichoderma* sp.0018 fermentation broth. Similarity, during co-cultivation of *Lentinula edodes* with *Trichoderma*, mycelium assemblage is also observed, which has been verified that the barrages characterized by brown antagonism lines are beneficial for resisting *Trichoderma* invasion and for defending territories in the contact zones [55]. Similar morphological changes were also reported in *P. ostreatus* [56] and *S. commune* [57]. Furthermore, the Gα has been verified to increase the ability to defend against pathogens in plants [58]. Hence, the results showed that the antagonism line regulated by *FfGa1* might have positive effects on enhancing the resistance of *F. filiformis* mycelia to biotic stress.

Generally, the cell wall is regarded as the first defensive barrier to biotic and abiotic stress in fungi [59]. Chitin is a core component of the inner cell wall, hydrophobins are a key class of cell wall proteins, and both of them are affected by Gα subunits [39,60]. As downstream genes related to the signaling pathway, the deletion of *Chs5* resulted in the abnormal morphology of hyphae and decreased the tolerance to heat stress, cell wall-perturbing compounds, and the deletion of *chs7* in *Metarhizium acridum* [61]; in contrast, the disruption of *Hyd1* increased the adaptability to thermal stress in *Beauveria bassiana* [62], and showed a high ability to maintain cell wall integrity in *Aspergillus fumigatus* [63]. In this study, most *Chs* gene expression levels were increased in OE transformants, though unlike *Chs* genes, the expression patterns of *Hyds* did not exhibit apparent regularity. *Hyd1* mRNA levels were reduced in OE transformants. As mentioned above, the data from the comparison analysis of phenotype traits and RNA quantification showed that *FfGa1* enhanced the cell wall integrity and biotic stress resistance of *F. filiformis* mycelia.

## 5. Conclusions

In summary, our results have shown that *FfGa1* plays a crucial positive role in cell wall integrity maintenance and protection against mycoparasite *Trichoderma*, as well as thermal stress in *F. filiformis*. In addition, we established that *FfGa1* increased hyphal extension and regulated the expression of the hydrophobin and chitin synthetase genes. This study is beneficial for exploring the role of Gα signaling pathways in macro-fungi and provides a suitable target point for the improved production of edible fungi.

## Data Availability

Not applicable.

## Supplementary Materials

The following are available online at https://www.mdpi.com/2309-608X/8/4/401/s1, Figure S1: PCR confirmation of the OE and RNAi transformants, Figure S2: Effect of fermentation broth of *Trichoderma* sp. 0018 on mycelium growth rate in WT, Table S1: All primers used in study, Table S2: Insert fragment information of four *Flammulina filiformis* transformants.
