# Supplementary material for: FFGA1 Protein Is Essential for Regulating Vegetative Growth, Cell Wall Integrity, and Protection against Stress in Flammunina filiformis
Source: J Fungi (Basel). 2022 Apr 14;8(4):401. doi: 10.3390/jof8040401 (PMC9030616; doi:10.3390/jof8040401)
Supplement: Supplementary file 1 [file jof-08-00401-s001.zip › jof-1676105-supplementary.pdf]

Corresponding PCR fragments band size (FfGa1-OE 1891 bp, FfGa1-RNAi 660 bp) were observed in the selected transformants (OE1, OE2, Ri1, and Ri2), suggesting that the target gene fragments were stably inserted in the WT strain.

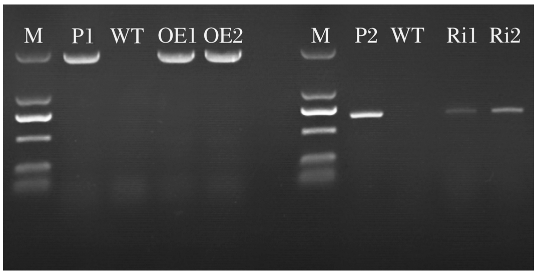

Figure S1 PCR confirmation of the OE and RNAi transformants. M represents DNA Marker DL2000; WT represents wild-type *F. filiformis* L11 strain; P1, represents plasmid FfGa1-OE; P2, represents plasmid FfGa1-RNAi.

The inhibition rate of WT cultured on PDA medium supplemented with 50% V/V *Trichoderma* spp.0018 culture broth was almost 37%, while, T5 with 75%V/V *Trichoderma* spp.0018 was too high, so we selected T4 with 50%V/V *Trichoderma* spp.0018 culture broth as treatment condition for further study.

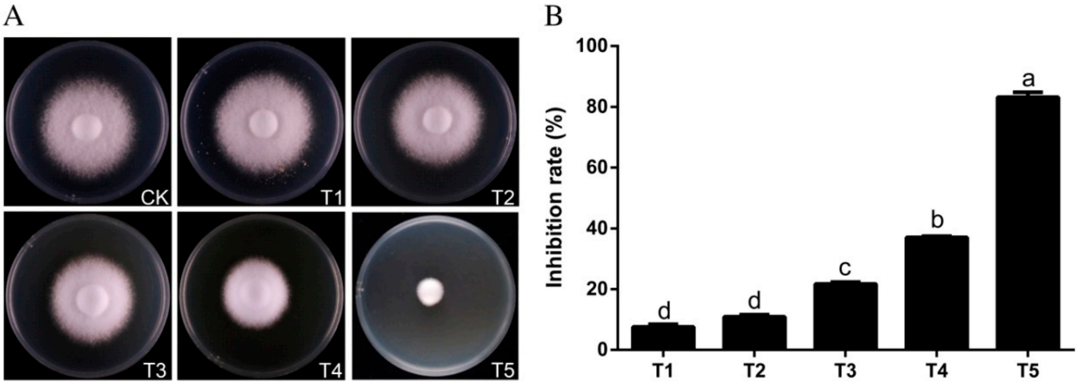

Figure S2. Effect of fermentaion broth of *Trichoderma* sp. 0018 on mycelium growth rate in WT. (A) Colony morphology of WT on PDA medium supplemented with *Trichoderma* sp.0018 fermentation broth. **(B)** Mycelial growth inhibition ratio of WT on PDA medium supplemented with *Trichoderma* sp.0018 fermentation broth CT represents PDA; T1: PDA with *Trichoderma* sp.0018 mycilium (0.5%); T2: PDA with *Trichoderma* sp.0018 fermentation broth (12.5%, v/v); T3: PDA with *Trichoderma* sp.0018 fermentation broth (25%, v/v); T4: PDA with *Trichoderma* sp.0018 fermentation broth (50%, v/v); T5: PDA with *Trichoderma* sp.0018 fermentation broth (75%, v/v). The letters "a"," b", "c" and "d" indicate statistically significant difference at 0.05 level between samples. Bars with no common letters are significantly different ($p < 0.05$).

Table S2 Insert fragment information of four *Flammulina filiformis* transformants

Strain	T-DNA site in vector	Location in genome	disrupted gene	Prediction protein	Sequence depth of genome	Sequence depth of insert fragment	Copy number*
OE1	5592-9902	Chr04: 1962738-1963193	gene205931	RNI-like protein [Hymenopellis radicata] (0, 62%)	157	131	1
OE2	5591-9717	Chr04: 19533323-1953745	gene205925	hypothetical protein BDZ89DRAFT_1127734 [Hymenopellis radicata](7e-79, 66%)	189	146	1
Ri1	5823-9222	Chr08: 1450860-1450914	gene210615	hypothetical protein ARMGADRAFT_1164320 [Armillaria gallica](1e-13,32%)	188	153	1
Ri2	5928-9223	Chr08: 1500978-1502687	gene210626	chondroitin AC/alginate lyase [Hymenopellis radicata](0,79%)	152	130	1

*: Sequence depth of insert fragment / Sequence depth of genome
